# Soft Robots with Cy5: An “Intake and Work” Imaging Technique for Intraoperative Navigation of Gastric Lesion

**DOI:** 10.34133/cbsystems.0212

**Published:** 2025-04-11

**Authors:** Lifeng He, Yu Pan, Wei Jin, Rong Tan, Yanan Xue, Danying Sun, Jingyu Zhang, Pingyu Xiang, Qin Fang, Yue Wang, Rong Xiong, Haojian Lu, Songmei Lou

**Affiliations:** ^1^ Department of General Surgery, Sir Run Run Shaw Hospital, School of Medicine, Zhejiang University, Hangzhou 310000, China.; ^2^ Cheng Kar-Shun Robotics Institute (CKSRI), Hong Kong University of Science and Technology, Hong Kong 999077, China.; ^3^ State Key Laboratory of Industrial Control and Technology, Zhejiang University, Hangzhou 310027, China.; ^4^ Institute of Cyber-Systems and Control, the Department of Control Science and Engineering, Zhejiang University, Hangzhou 310027, China.; ^5^ Stomatology Hospital, School of Stomatology, Zhejiang University School of Medicine, Clinical Reach Center for Oral Disease of Zhejiang Province, Key Laboratory of Oral Biomedical Reach of Zhejiang Province, Cancer Center of Zhejiang University, Hangzhou 310006, China.

## Abstract

Locating tumors during laparoscopic surgery for early gastric cancers poses an important challenge because they lack involvement with the serosal layer and remain invisible within the peritoneal cavity. To address this issue, various techniques such as preoperative dye injection and magnetic clip detection systems have been introduced to aid in intraoperative tumor localization. However, these existing techniques are often intricate and lack intuition and endurance. In this study, we propose a novel approach utilizing fluorescent soft robots to accurately locate tumors within the stomach. The methodology involved placing a metal clip at the tumor site, followed by administering several soft robots labeled with Cy5. These soft robots were designed to autonomously converge around the metal clip. To validate their efficacy, we conducted animal experiments by implanting clips into the stomachs of rats and subsequently administering capsules containing the soft robots. By detecting the resulting fluorescence, we successfully identified the location of the clips within the stomach. Our findings indicate that these soft robots hold great promise as a viable alternative for localizing gastric lesions during laparoscopic surgery, which has better persistence and intuitiveness than other markup methods. Their implementation could significantly enhance the accuracy and efficiency of tumor identification in a technologically advanced and clinically accessible manner.

## Introduction

Gastric cancer, a prevalent malignant tumor of the digestive system worldwide, has seen an increase in early diagnoses due to the implementation of endoscopic screening systems in recent years [1,2]. This advancement has led to improved surgical outcomes for gastric cancer patients. Laparoscopic surgery for early gastric cancer, initially introduced by Kitano in 1994, has gained global popularity due to its notable short-term benefits and comparable oncological prognosis to open surgery [3–6]. However, accurately locating early gastric cancer during laparoscopic surgery remains a challenge, as these tumors are limited to the mucous and submucosal membranes, making them undetectable through gross analysis of the serosa layer in the intraperitoneal view.

In clinical practice, intraoperative endoscopy examination is commonly employed for precise lesion marking. However, this approach has certain drawbacks, such as the need for an experienced endoscopist and the potential adverse impact on the surgical procedure due to air inflation. Consequently, surgeons often schedule a second endoscopy the day before surgery. Dye tattooing is a commonly used technique during the second endoscopy to mark the tumor. Various dyes, including methylene blue, indigo carmine, toluidine blue, isosulfan blue, hematoxylin and eosin (H&E), autologous blood, indocyanine green (ICG), and India ink, are currently employed for this purpose [7–9]. The dye remains visible at the tattooed location for 12 h or longer. The advantage of this method is that the dye can be directly visualized with the naked eye during laparoscopic surgery. However, the spreading and eventual disappearance of the dye may lead to imprecise resection margins and potential tumor dissemination. Additionally, tissue swelling and inflammatory reactions can increase surgical difficulty. Therefore, dye tattooing is typically performed as close to the operation as possible to minimize adverse effects. In clinical practice, endoscopists often employ the placement of a metal clip to mark suspected lesions after a biopsy. This approach aims to eliminate the need for additional endoscopy examinations. Various techniques have been reported to detect the clip and accurately locate the tumor, including endoscopic clips integrated with light-emitting diodes (LEDs), endoscope clips utilizing radiofrequency identification, and endoscope clips containing ICG dyes [10–14]. However, these techniques possess certain limitations. For instance, devices requiring a power supply can be cumbersome, and the presence of electromagnetic interference can interfere with their functionality. Although metal clips incorporating ICG dyes eliminate the need for a power supply and are immune to electromagnetic interference, the duration of fluorescence dissipation must be taken into consideration when installing the metal clips preoperatively.

**Fig. 1.**
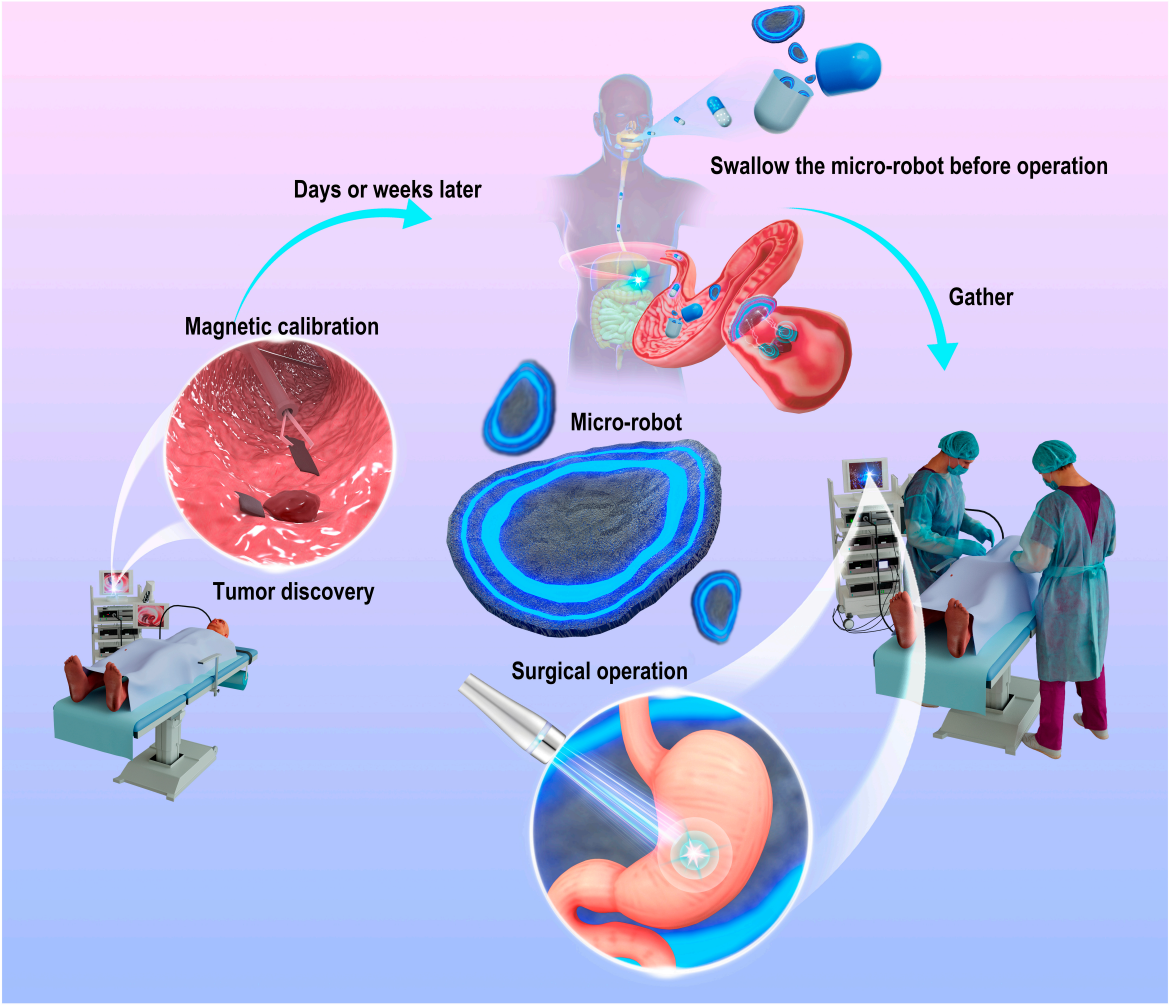
Schematic of soft robots with Cy5 for intraoperative navigation of gastric lesion. A magnetic marker is installed with the metal clip at the suspected lesion during the endoscopic examination. Before operation, a capsule containing the soft robots with Cy5 is taken. The soft robots gather and adhere to preinstalled metal clip under the magnetic field. The lesion is lighted up and can be easily figured out under NIR light during the operation.

Therefore, the design of a highly efficient marker that can be released at the suspected lesion site after a biopsy, thereby eliminating the requirement for additional endoscopic examinations, becomes crucial. This marker clip should be accurately and instantly detectable while also being easily accessible. The detection system itself should be stable, minimize time consumption, and be easy to operate. Soft robots, which have been developed for biomedical applications, including drug delivery, navigation in minimally invasive surgery, and manipulation of the cellular microenvironment, hold great potential [15–18]. In this study, we present the design of a fluorescent magnetic micro-robot labeled with Cy5. This micro-robot can be transported to the stomach using a capsule and adhere to the preinstalled metal clip, thus illuminating the lesion under near-infrared (NIR) light (refer to Fig. 1 for visualization).

## Materials and Methods

### Cell culture of liver cancer cell lines

Liver cancer cell line (HCCLM3) was purchased from the American Type Culture Collection (Manassas, VA, USA) and cultured in accordance with the recommended guidelines.

### Cell viability test

Cells were seeded in 96-well plates in replicates of 3. After incubation with Cy5 soft robots as the experimental group, the cells were cultured separately as the control group and cell viability analysis was performed using the CellTiter 96 AQueous One Solution Cell Proliferation Assay (MTS) assay. MTS (Promega, catalog no. G3580) was added to each well, and the absorbance values were measured at a wavelength of 490 nm by spectrophotometry.

### Cell cycle assay

Cells were seeded in 6-well plates and mock-treated or treated with Cy5 soft robots. Cell cycle was determined using the Cell Cycle Kit (Multi Sciences, Hangzhou, China).

### Animal surgery

Sprague–Dawley (SD) rats were purchased from Shanghai Slack Laboratory Animal Co. Ltd. (China) and housed under standard pathogen-free (SPF) conditions. The study was approved by the local authorities (Zhejiang University Laboratory Animal Research Center, China). SD rats were anesthetized with 1% pentobarbital sodium (P-010, Merck, China) at a dose of 4 ml/kg. Metal clips were installed on the rat stomach during surgery. Then, a capsule containing Cy5 soft robots was fed following operation. Fluorescence was detected using the IVIS Lumina optical imaging system (PerkinElmer, USA) for small animals in vivo. To verify the safety of the Cy5 soft robots in vivo, we measured serum glutamic-pyruvic transaminase (ALT), glutamic oxalacetic transaminase (AST), and urea nitrogen (UN) levels 24 h later in SD rats treated with surgically unimmobilized Cy5 soft robots in the stomach as an experimental group and SD rats treated with surgically immobilized robot as a control group.

### Statistical analysis

Statistical analysis was conducted using GraphPad Prism 8. Quantitative data between groups were compared using a *t* test or pair *t* test. Data were presented as the mean ± SEM from at least 3 independent experiments. In all results, a 2-tailed *P* value of <0.05 was considered statistically significant.

## Results

### Design of Cy5 soft robots and magnetic field

**Fig. 2.**
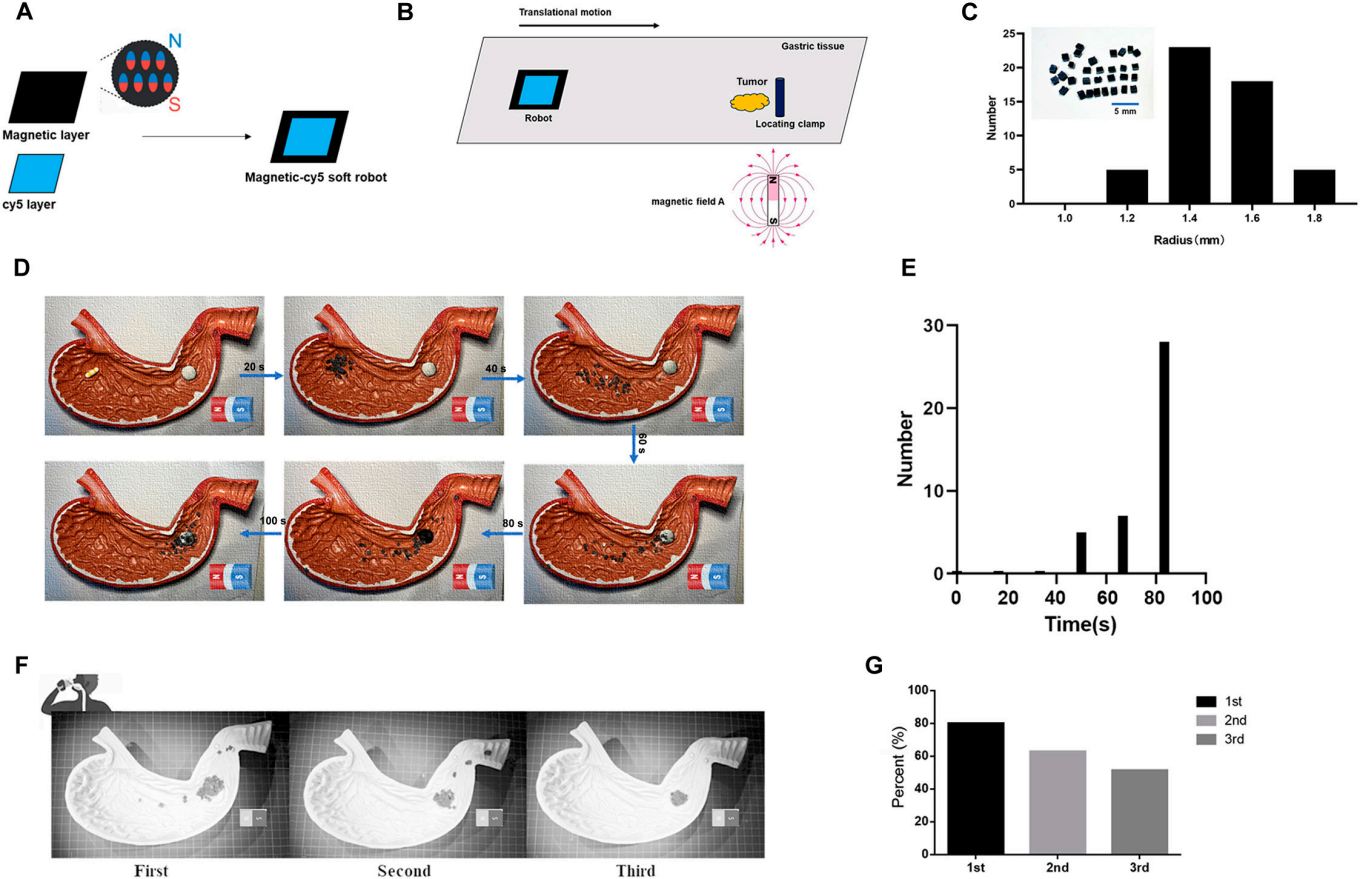
Fabrication of soft robots with Cy5. (A) Design of Cy5 soft robots. (B) Design of Cy5 soft robots and magnetic field. (C) Size distribution of soft robots. (D) Orientation movement of soft robots on the stomach model under the magnetic field. (E) Number of soft robots that successfully reached the preset destination. (F) Hose stream experiment on the stomach model to test the adhesion ability of soft robots. (G) Number of soft robots remaining on the marker after 3 times of hose stream.

The fabrication of the Cy5 soft robots was carried out using previously reported methods, such as lithography, doped silicone molding, and direct laser writing [19]. In this study, the fabrication process of the soft robots followed a specific procedure. Initially, a mixture of silicone gel and iron powder was prepared in a mass ratio of 1:1. This mixture was then poured uniformly onto a 3-dimensional (3D)-printed mold. Subsequently, the mold was subjected to a uniform magnetic field, which facilitated the rearrangement of the iron particles within the silicone gel. Following this step, Cy5-labeled silicone gel was poured onto the mold, and once fully set, the silicone gel structure was carefully removed from the mold. Finally, the structure was cut into the desired shape, completing the fabrication of the Cy5 soft robots (Fig. 2A). In clinical applications, NIR fluorescent materials play a crucial role, particularly in navigation during minimally invasive surgeries [7,20,21]. In this study, Cy5 was chosen as the fluorescent material instead of the conventional ICG. While ICG theoretically exhibits better fluorescence penetration ability than Cy5, previous studies have shown that Cy5 actually offers several advantages over ICG, particularly in terms of brightness. These differences in performance can be attributed to variations in their respective quantum yields [22]. Optical imaging in the NIR range holds great promise for clinical applications due to its ability to reduce light scattering in tissues and provide increased penetration depth compared to the visible range. Therefore, the utilization of Cy5 as a fluorescent material in this study represents a highly promising approach for clinical applications.

In addition, we design a magnetic ring, which was prepared by mixing silica gel with magnetic powder at a mass ratio of 1:1. This mixture is then poured evenly over the 3D printing mold. When we find the lesion during the first gastroscopy, this magnetic ring will be fixed around the lesion with a titanium clamp, thus forming an internal magnetic field. The soft robot will enter the patient’s stomach cavity in the form of a capsule and be released in the stomach. The external magnetic field will guide the robot in the direction of the lesion, and the internal magnetic field will attract the robot to accumulate around the lesion by applying magnetic gradient force Fm (Fig. 2B).

The soft robots have been miniaturized to millimeter scales, making them well suited for minimally invasive medical and surgical applications. The fabrication process for these soft robots is relatively simple, and they can be easily cut into appropriate shapes. The sizes of the soft robots are uniform, typically ranging from 1.2 to 1.6 mm (Fig. 2C). In this study, the orientation movement of the soft robots plays a crucial role. Once the capsule containing the soft robots reaches the designated position and the soft robots are released, the soft robots quickly organize themselves into a powerful swarm by moving and rotating rapidly under the influence of a magnet. This enables the soft robots to navigate along a predetermined route on a damp stomach model, as depicted in Fig. 2B. As demonstrated in Fig. 2D, all the soft robots successfully reached the preset destination within 100 s, indicating their acceptable velocity. The peristalsis movements of the stomach necessitate tight adhesion of soft robots to the metal clip in order to accurately illuminate the lesion. To evaluate their adhesion ability, we simulated intragastric conditions using a hose stream, as depicted in Fig. 2E. The results indicate that the majority of soft robots remained tightly adhered to the metal clip even after being subjected to 3 rounds of hose streaming. Specifically, 80.0% of the soft robots remained after the first round, 62.9% remained after the second round, and 54.3% remained after the third round (Fig. 2F and G).

### Cytotoxicity and fluorescent dissipation of Cy5 soft robots

**Fig. 3.**
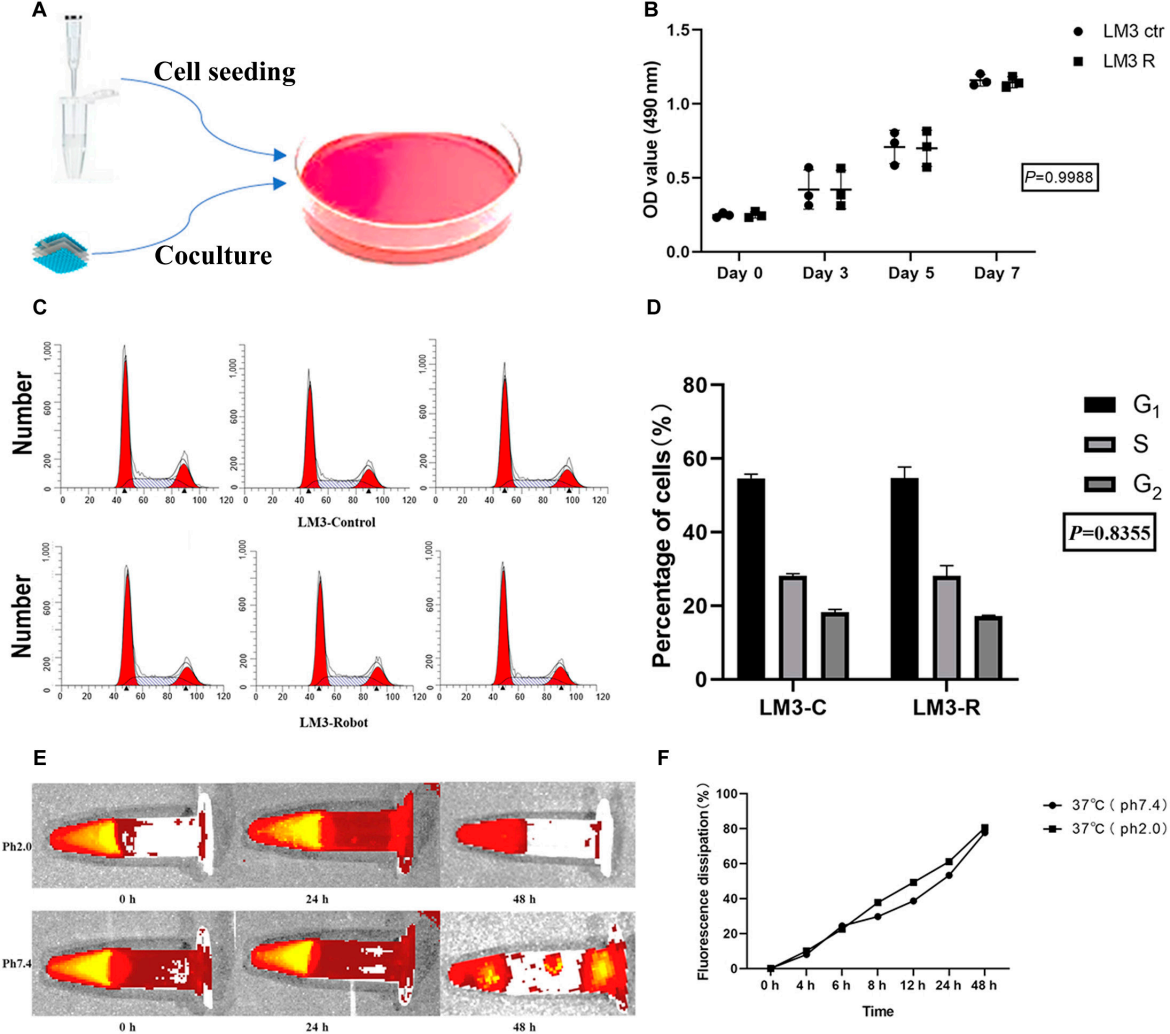
Cytotoxicity and fluorescent dissipation of Cy5 soft robots in vitro. (A) Schematic of cell coculture. (B) Soft robots with Cy5 did not significantly decrease cell viability in MTS analysis. (C) Soft robots with Cy5 did not significantly alter cell cycle. (D) Comparison of different cell cycle phase between soft robots with Cy5 and control group. (E) Comparison of different cell cycle phase between soft robots with Cy5 and control group. (F) Fluorescent dissipation of Cy5 soft robots.

To evaluate the cytotoxicity of the soft robots, in vitro experiments were conducted by coculturing them with liver cancer cells, as depicted in Fig. 3A. The cytotoxicity of the as-prepared Cy5 soft robots toward liver cancer cells was subsequently assessed using the MTS assay. The results revealed that the as-prepared soft robots did not exhibit any significant negative effects on cellular viabilities within a 1-week period (Fig. 3B). The addition of the soft robots to the cell cultures did not hinder cell proliferation (Fig. 3C) nor alter the distribution of cell cycle phases (Fig. 3D). These findings suggest that the soft robots exhibit good biocompatibility, making them suitable for therapeutic applications.

Because of their embedding in silicone gel, the stability of Cy5 under acidic conditions is maintained. As shown in Fig. 3E, the fluorescent signal emitted by the Cy5 soft robots was detected in vitro for 48 h under acidic conditions with a pH of 2.0, albeit slightly weaker compared to normal conditions with a pH of 7.0. The rate of fluorescent dissipation of the Cy5 soft robots at pH 2.0 was comparable to that at pH 7.0 (*P* > 0.05) over the 48-hour period at 37°C (dissipation rate at 48 h, soft robots group: 77.7% versus control group: 80.5%) (Fig. 3F). These results indicate that the Cy5 soft robots are capable of emitting an infrared fluorescent signal consistently even in the acidic environment characteristic of the stomach.

### Fluorescence intensity measurement of Cy5 soft robots in vivo

**Fig. 4.**
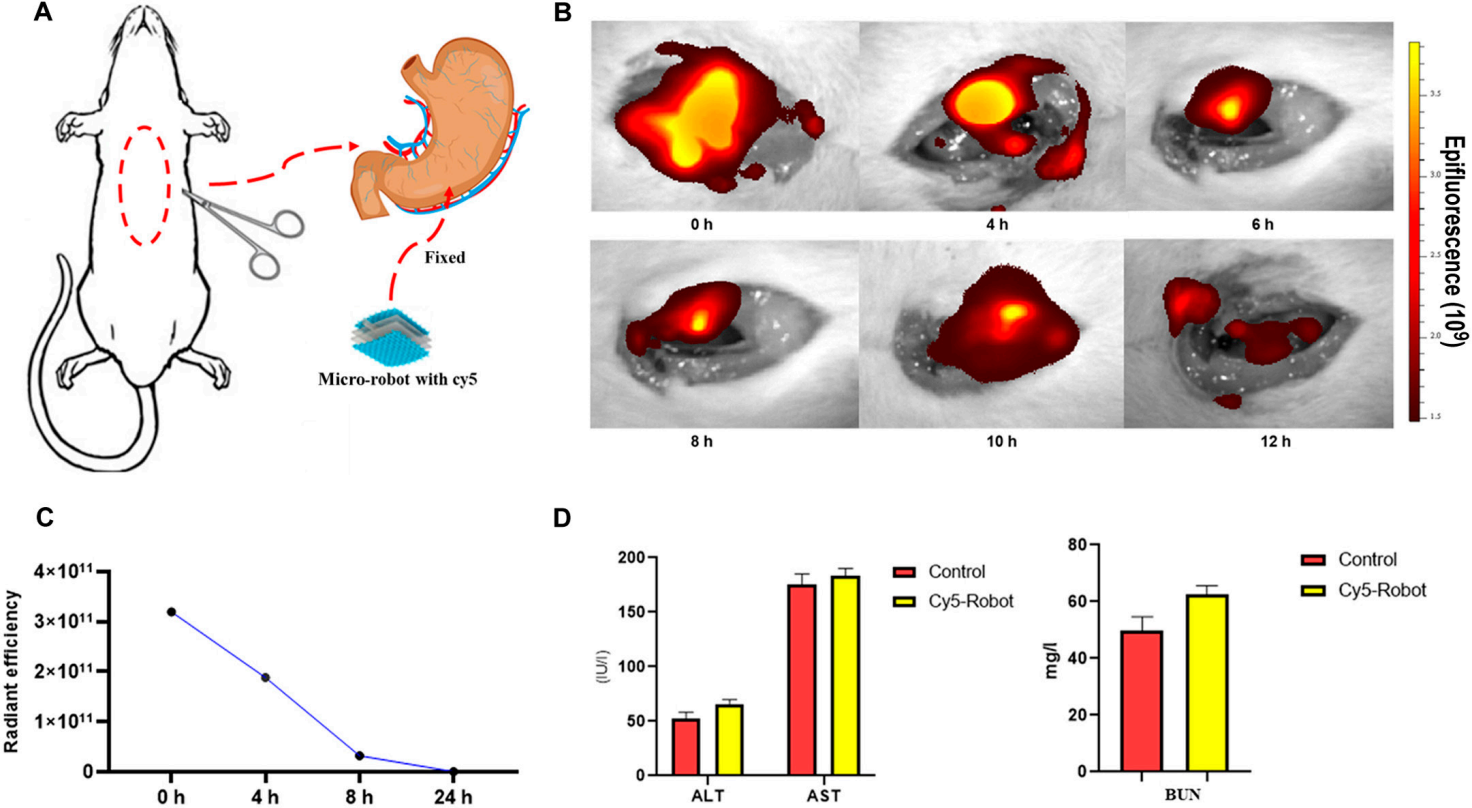
Fluorescence intensity measurement and effects on hepatic and renal function of Cy5 soft robots in vivo. (A) Schematic of animal surgery by suturing soft robots on the rat stomach. (B) Fluorescence signal of soft robots with Cy5 in vivo. (C) Fluorescence intensity of soft robots with Cy5 in 24 h. (D) Blood sample analysis of AST, ALT, and BUN from soft robots with Cy5 and control group.

The fluorescence intensity of the Cy5 soft robots in an in vivo setting was assessed by suturing them onto the stomach for detection purposes during an operation (Fig. 4A). As shown in Fig. 4B, the Cy5 soft robots exhibited a high fluorescence signal in vivo, resulting in a distinct image. The fluorescence signal could be detected within 24 h, indicating the potential feasibility of this approach in clinical practice (Fig. 4C). To evaluate the potential adverse effects of the soft robots on hepatic and renal function, blood samples were collected from the rats. The levels of AST (aspartate aminotransferase), ALT (alanine aminotransferase), and BUN (blood urea nitrogen) were measured. The results showed no significant difference in these parameters between the soft robots group and the control group (*P* > 0.05). This suggests that the soft robots did not induce any serious adverse effects on hepatic and renal function (Fig. 4C).

### Imaging of clip marker

**Fig. 5.**
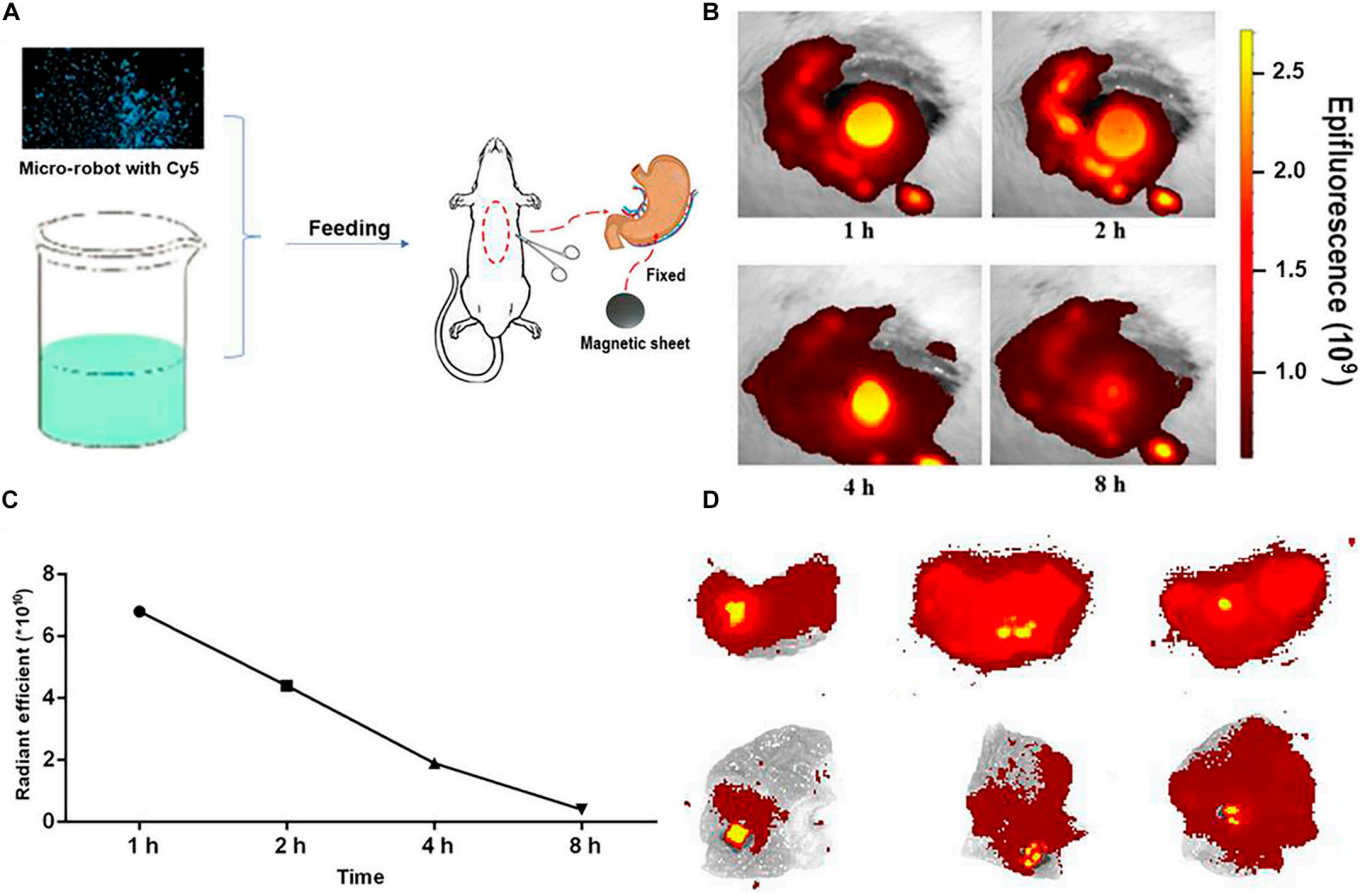
Fluorescence imaging of metal clip using the Cy5 soft robots. (A) Schematic illustration of fluorescence imaging of metal clip in the stomach. (B) Metal clip was lighted up by feeding a capsule containing Cy5 soft robots in vivo. (C) Fluorescence imaging of metal clip maintained for 8 h. (D) The soft robots accurately located the lesion in the stomach including antrum, body, and fundus.

To illustrate the process, Fig. 5A provides a schematic representation of the clip marker imaging conducted in the rat stomach. An operation was performed on the rat stomach, and a metal clip with a magnet marker was installed. Subsequently, the rat was fed a capsule containing Cy5 soft robots. Once inside the stomach, the capsule disintegrated, releasing the soft robots. Through rapid rotation of the magnet, the soft robots moved toward and adhered to the metal clip in vitro. Under NIR light, the metal clip with the magnet marker immediately emitted a high-resolution fluorescence signal with an extremely low background signal for up to 8 h (dissipation rate at 8 h: 87.6%) (Fig. 5B and C). This demonstrates the sustained visibility and accuracy of the metal clip with the magnet marker over time. Metal clips were installed in various regions to assess the soft robots’ ability to locate lesions in different areas of the stomach. As depicted in Fig. 5D, the soft robots successfully and accurately located the lesions in different parts of the stomach, including the antrum, body, and fundus. This highlights the capability of the soft robots to precisely identify and localize lesions in different areas of the stomach.

## Discussion

Soft robots offer significant advantages over several techniques that have been explored in preclinical trials for preoperative localization of stomach lesions. For instance, intraoperative laparoscopic ultrasonography, as proposed by Matsuda et al. [23], requires surgeons with extensive expertise to precisely locate the small metal clip during surgery using a specialized ultrasound instrument. However, this technique can be challenging, particularly for tumors located in the posterior wall of the stomach due to the presence of intragastric gas. Another technique involves using a specially manufactured magnetic clip and a magnetic marker clip detection system to locate tumors during surgery [24]. While this system allows for easy detection of the clips without any training or learning curve, it does not provide precise tumor localization. Reported errors in locating lesions using this method ranged from an average of approximately 5.7 mm to a maximum of approximately 10.1 mm.

Radio-frequency identification (RFID) detection is another approach where a clip containing a unique RFID tag is applied before surgery and detected in real time during laparoscopic surgery using a novel detection system. While this technique enables high detection accuracy, the wire connection in the system is not sturdy and tends to wobble, posing limitations. To overcome the disadvantages of dye injection on the wall of hollow viscera, a fluorescent clip has been developed, which can be visualized by a laser and a digital charge-coupled device (CCD) camera during surgery. However, the detection accuracy may decrease due to reduced fluorescence transmittance or signal attenuation if the early gastric cancer (EGC) is located in a thick stomach wall.

In contrast, the magnetic micro-robot with Cy5 offers ease of operation by simply taking a capsule, eliminating the need for a complex system. A swarm of soft robots can emit a high fluorescent signal intensity and maintain it for a sufficient duration for operative preparation. The present study demonstrates the technical feasibility of using soft robots with Cy5 for preoperative localization of stomach lesions, providing a promising alternative for laparoscopic surgery.

## Conclusion

In this study, the designed fluorescent magnetic micro-robot offers several advantages for marking gastric lesions using approved metal clips. The fabrication process is simple, allowing for easy production of the soft robots. The soft robots demonstrate excellent movement ability, enabling them to navigate within the stomach and reach the desired locations. Additionally, they exhibit strong adhesion to the metal clips, ensuring accurate marking of the lesions. One notable advantage of the soft robots is their ability to function effectively in an acidic environment, such as the stomach. This characteristic is important as it allows the soft robots to maintain their functionality and fluorescence even in the presence of gastric acid. Another significant benefit is the long-lasting fluorescence of the soft robots, which can be maintained for more than 8 h. This duration meets the requirements for preoperative fasting, ensuring that the fluorescence signal remains visible and useful throughout the preoperative period. The combination of these advantages makes the fluorescent magnetic micro-robot a promising alternative for instantly available localization of gastric lesions. By leveraging their simple fabrication process, excellent mobility, strong adhesion, and ability to operate in an acidic environment while maintaining long-lasting fluorescence, these soft robots offer a potential solution for efficient and accurate localization of gastric lesions in clinical practice.

## Data Availability

The data are available from the authors upon a reasonable request.

## References

[1] Sung H, Ferlay J, Siegel RL, Laversanne M, Soerjomataram I, Jemal A, Bray F. Global cancer statistics 2020: GLOBOCAN estimates of incidence and mortality worldwide for 36 cancers in 185 countries. CA Cancer J Clin. 2021;71(3):209–249. 33538338 10.3322/caac.21660

[2] Necula L, Matei L, Dragu D, Neagu AI, Mambet C, Nedeianu S, Bleotu C, Diaconu CC, Chivu-Economescu M. Recent advances in gastric cancer early diagnosis. World J Gastroenterol. 2019;25(17):2029–2044. 31114131 10.3748/wjg.v25.i17.2029 PMC6506585

[3] Kitano S, Yasuda K, Shiraishi N. Laparoscopic surgical resection for early gastric cancer. Eur J Gastroenterol Hepatol. 2006;18(8):855–861. 16825901 10.1097/00042737-200608000-00008

[4] Emile SH, Barsom SH. Short-term outcomes of single-incision compared to multi-port laparoscopic gastrectomy for gastric cancer: A meta-analysis of randomized controlled trials. Laparosc Endosc Robot Surg. 2023;6(4):127–133.

[5] Takiguchi S, Fujiwara Y, Yamasaki M, Miyata H, Nakajima K, Sekimoto M, Mori M, Doki Y. Laparoscopy-assisted distal gastrectomy versus open distal gastrectomy. A prospective randomized single-blind study. World J Surg. 2013;37:2379–2386. 23783252 10.1007/s00268-013-2121-7

[6] Kim YW, Baik YH, Yun YH, Nam BH, Kim DH, Choi IJ, Bae JM. Improved quality of life outcomes after laparoscopy-assisted distal gastrectomy for early gastric cancer: Results of a prospective randomized clinical trial. Ann Surg. 2008;248(5):721–727. 18948798 10.1097/SLA.0b013e318185e62e

[7] Chen QY, Xie JW, Zhong Q, Wang JB, Lin JX, Lu J, Cao LL, Lin M, Tu RH, Huang ZN, et al. Safety and efficacy of indocyanine green tracer-guided lymph node dissection during laparoscopic radical gastrectomy in patients with gastric cancer: A randomized clinical trial. JAMA Surg. 2020;155:300–311. 32101269 10.1001/jamasurg.2019.6033

[8] Tokuhara T, Nakata E, Tenjo T, Kawai I, Satoi S, Inoue K, Araki M, Ueda H, Higashi C. A novel option for preoperative endoscopic marking with India ink in totally laparoscopic distal gastrectomy for gastric cancer: A useful technique considering the morphological characteristics of the stomach. Mol Clin Oncol. 2017;6:483–486. 28413653 10.3892/mco.2017.1191 PMC5374967

[9] Ushimaru Y, Omori T, Fujiwara Y, Yanagimoto Y, Sugimura K, Yamamoto K, Moon JH, Miyata H, Ohue M, Yano M. The feasibility and safety of preoperative fluorescence marking with indocyanine green (ICG) in laparoscopic gastrectomy for gastric cancer. J Gastrointest Surg. 2019;23(3):468–476. 30084063 10.1007/s11605-018-3900-0

[10] Sugiyama M, Nagao Y, Uehara H, Kagawa M, Shin Y, Shiokawa K, Ota M, Akahoshi T, Morita M. Wireless light-emitting marker using magnetic field resonance for laparoscopic gastrointestinal surgery. Surg Laparosc Endosc Percutan Tech. 2021;31:778–781. 33734210 10.1097/SLE.0000000000000929

[11] Wada Y, Miyoshi N, Fujino S, Ohue M, Yasui M, Takahashi Y, Takahashi H, Nishimura J, Takenaka Y, Saso K, et al. New marking method involving a light-emitting diode and power source device to localize gastrointestinal cancer in laparoscopic surgery. Sci Rep. 2019;9:5485. 30940902 10.1038/s41598-019-41981-w PMC6445110

[12] Lee KM, Min JS, Choi WJ, Ahn JW, Yoon SW, Kim YJ. Radioactive synthesis of tau PET imaging agent ^18^F-AV-1451 and its role in monitoring the progression of Alzheimer’s disease and supporting differential diagnosis. Surg Endosc. 2021;35(2):139–147. 33460010 10.1007/s12149-020-01566-4

[13] Hyun JH, Kim SK, Kim KG, Kim HR, Lee HM, Park S, Kim SC, Choi Y, Sohn DK. A novel endoscopic fluorescent band ligation method for tumor localization. Surg Endosc. 2016;30:4659–4663. 26895900 10.1007/s00464-016-4785-1

[14] Barberio M, Pizzicannella M, Spota A, Ashoka AH, Agnus V, Al Taher M, Jansen-Winkeln B, Gockel I, Marescaux J, Swanström L, et al. Preoperative endoscopic marking of the gastrointestinal tract using fluorescence imaging: Submucosal indocyanine green tattooing versus a novel fluorescent over-the-scope clip in a survival experimental study. Surg Endosc. 2021;35:5115–5123. 32989536 10.1007/s00464-020-07999-2 PMC8346416

[15] Zhang W, Deng Y, Zhao J, Zhang T, Zhang X, Song W, Wang L, Li T. Amoeba-inspired magnetic venom microrobots. Small. 2023;19(23):2207360. 10.1002/smll.202207360 36869412

[16] Li T, Yu S, Sun B, Li Y, Wang X, Pan Y, Song C, Ren Y, Zhang Z, Grattan KTV, et al. Bioinspired claw-engaged and biolubricated swimming microrobots creating active retention in blood vessels. Sci Adv. 2023;9(18):eadg4501. 37146139 10.1126/sciadv.adg4501 PMC10162671

[17] Yu S, Lia T, Jib F, Zhao S, Liu K, Zhang Z, Zhang W, Mei Y. Trimer-like microrobots with multimodal locomotion and reconfigurable capabilities. Mater Today Adv. 2022;14: Article 100231.

[18] Zhang W, Deng Y, Zhao J, Zhang T, Zhang X, Song W, Wang L, Li T. Propulsion gait analysis and fluidic trapping of swinging flexible nanomotors. ACS Nano. 2021;15(3):5118–5128. 33687190 10.1021/acsnano.0c10269

[19] Lu H, Zhang M, Yang Y, Huang Q, Fukuda T, Wang Z, Shen Y. A bioinspired multilegged soft millirobot that functions in both dry and wet conditions. Nat Commun. 2018;9(1):3944. 30258072 10.1038/s41467-018-06491-9 PMC6158235

[20] Qian X, Hu W, Gao L, Xu J, Wang B, Song J, Yang S, Lu Q, Zhang L, Yan J, et al. Trans-arterial positive ICG staining-guided laparoscopic liver watershed resection for hepatocellular carcinoma. Front Oncol. 2022;12: Article 966626. 35936704 10.3389/fonc.2022.966626 PMC9354495

[21] Maruri I, Pardellas MH, Cano-Valderrama O, Jove P, López-Otero M, Otero I, Campo V, Fernández R, Fernández-Fernández N, Sánchez-Santos R. Retrospective cohort study of laparoscopic ICG-Guided. Surg Endosc. 2022;36:8164–8169. 35486191 10.1007/s00464-022-09258-y

[22] Oh G, Cho HJ, Suh S, Lee D, Kim K. Multicolor fluorescence imaging using a single RGB-IR CMOS sensor for cancer detection with smURFP-labeled probiotics. Biomed Opt Express. 2020;11(6):2951–2963. 32637234 10.1364/BOE.391417 PMC7316003

[23] Matsuda T, Iwasaki T, Hirata K, Tsugawa D, Sugita Y, Ishida S, Kanaji S, Kakeji Y. Simple and reliable method for tumor localization during totally laparoscopic gastrectomy: Intraoperative laparoscopic ultrasonography combined with tattooing. Gastric Cancer. 2017;20:548–552. 27539582 10.1007/s10120-016-0635-z

[24] Yoon K, Kim KG, Chung JW, Lee WS. Clip-detector using a neodymium magnet to locate malignant tumors during laparoscopic surgery. Sensors. 2022;22(14):5404. 35891084 10.3390/s22145404 PMC9319524

