# Supplementary material for: Soft Robots with Cy5: An “Intake and Work” Imaging Technique for Intraoperative Navigation of Gastric Lesion
Source: Cyborg Bionic Syst. 2025 Apr 11;6:0212. doi: 10.34133/cbsystems.0212 (PMC11986207; doi:10.34133/cbsystems.0212)
Supplement: Supplementary 1 — Figs. S1 and S2 [file cbsystems.0212.f1.docx]

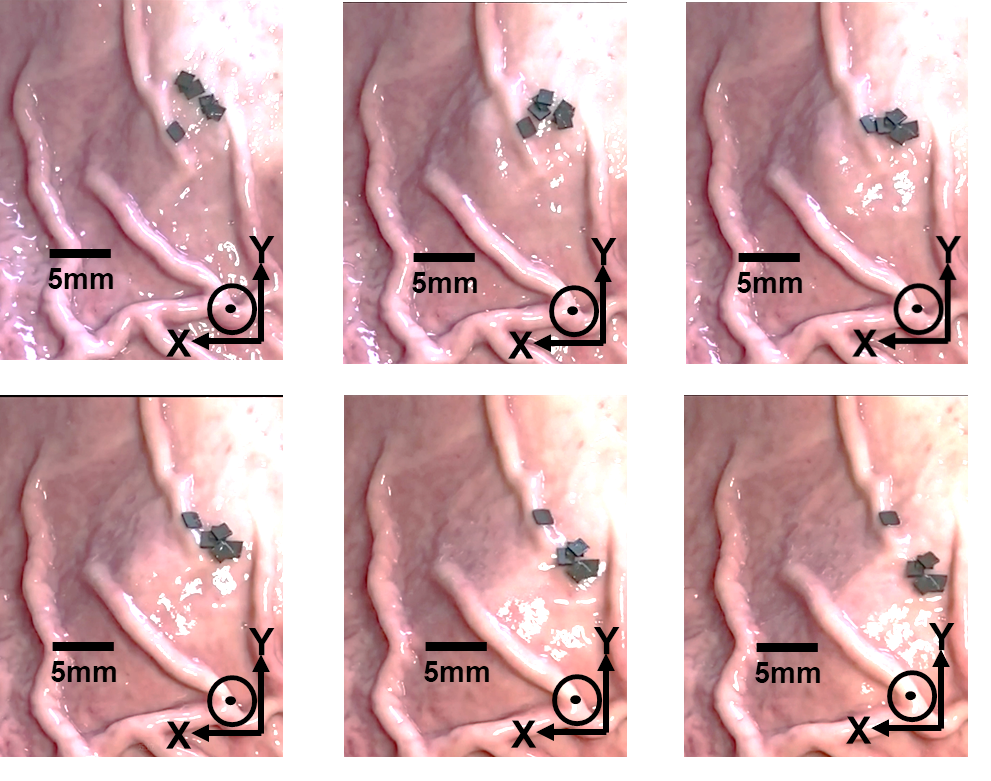


Supplementary Fig1. Motion diagram of a soft robot on the surface of gastric mucosa under the influence of an externally applied magnetic field A.


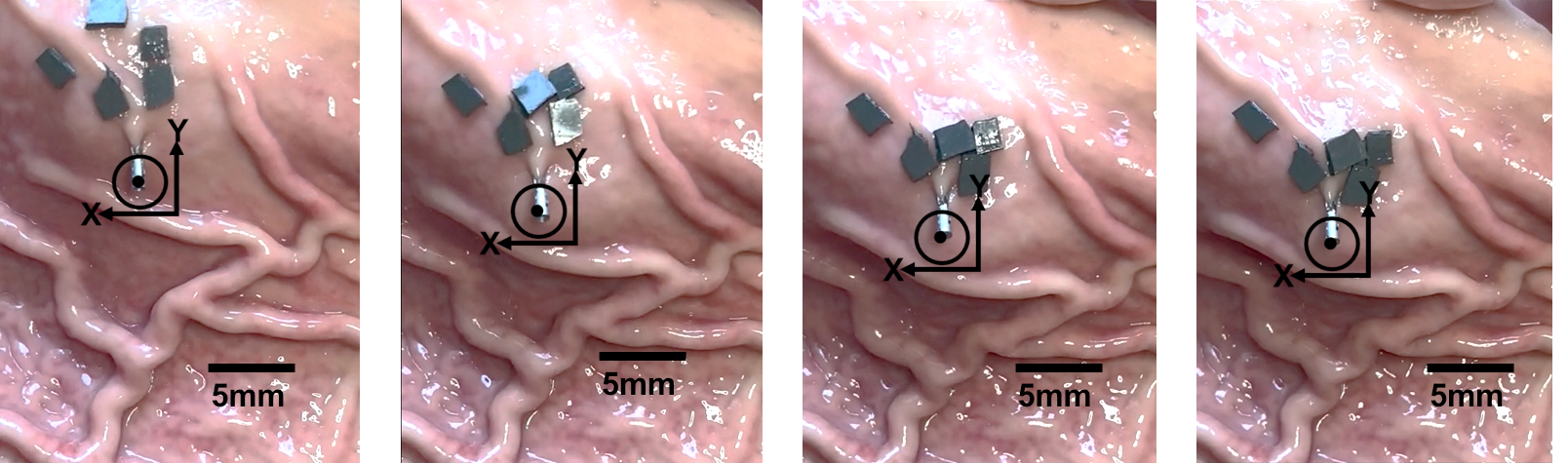


Supplementary Fig2. Motion diagram of a soft robot on the surface of gastric mucosa under the influence of an internal magnetic field B.
